# Intra-Individual Variability in Vagal Control Is Associated With Response Inhibition Under Stress

**DOI:** 10.3389/fnhum.2018.00475

**Published:** 2018-11-27

**Authors:** Derek P. Spangler, Katherine R. Gamble, Jared J. McGinley, Julian F. Thayer, Justin R. Brooks

**Affiliations:** ^1^Human Research & Engineering Directorate, U.S. Army Research Laboratory, Aberdeen, MD, United States; ^2^Department of Psychology, Towson University, Towson, MD, United States; ^3^Department of Psychology, The Ohio State University, Columbus, OH, United States

**Keywords:** heart rate variability, cognition, stress, intra-individual variability, response inhibition

## Abstract

Dynamic intra-individual variability (IIV) in cardiac vagal control across multiple situations is believed to contribute to adaptive cognition under stress; however, a dearth of research has empirically tested this notion. To this end, we examined 25 U.S. Army Soldiers (all male, *mean age* = 30.73, *standard deviation (SD)* = 7.71) whose high-frequency heart rate variability (HF-HRV) was measured during a resting baseline and during three conditions of a shooting task (training, low stress, high stress). Response inhibition was measured as the correct rejection (CR) of friendly targets during the low and high stress conditions. We tested the association between the SD of HF-HRV across all four task conditions (IIV in vagal control) and changes in response inhibition between low and high stress. Greater differences in vagal control between conditions (larger IIV) were associated with higher tonic vagal control during rest, and stronger stress-related decreases in response inhibition. These results suggest that flexibility in vagal control is supported by tonic vagal control, but this flexibility also uniquely relates to adaptive cognition under stress. Findings are consistent with neurobehavioral and dynamical systems theories of vagal function.

## Introduction

Cardiac vagal control—the parasympathetic modulation of cardiac function by the vagus nerve—is associated with the neural mechanisms of stress, cognition, and human performance (Porges, 2007; Thayer and Lane, 2009). Non-invasive metrics of vagal control (i.e., high-frequency heart rate variability, HF-HRV) may thus help monitor and/or predict human performance in high stress environments (Hoover et al., 2012; McDuff et al., 2014). However, this first requires clarification of the fundamental associations between cognition under stress and different aspects of vagal function, particularly in real-world contexts. With this aim, the current study investigated intra-individual variability (IIV; i.e., within-person differences between multiple situations) in vagal control, a theoretically important but understudied metric of vagal function, and its associations with response inhibition during ecologically valid stress.

### Stress and Cognition

Stress is an evolutionary adaptation involving physiological and psychological changes that defend the organism against potential harm (Cannon, 1929; McEwen and Sapolsky, 1995; McEwen, 1998). Stress enhances bottom-up defensive responses (e.g., heightened startle reflex; environmental scanning) that protect the organism from danger, but it impairs top-down cognition that is less critical for immediate defense (Öhman et al., 2001; Lang and Davis, 2006; Phelps, 2006; Eysenck et al., 2007). Minimizing the deleterious effects of stress on cognition is adaptive for daily activities that are interrupted by mild stressors. However, impaired cognition due to high stress (robust or salient threat) may be context-appropriate and even adaptive for survival. In particular, impairments to response inhibition (i.e., impaired suppression of inappropriate responses) may help express defensive behaviors that protect the organism against immediate threat (e.g., wartime scenarios; Nesse, 2005).

### Vagal Control: Links to Stress and Cognition

Multiple theoretical models have posited a link between vagal function, and context-appropriate responding, including changes in cognition during stress. Drawing from a dynamical systems view, both the Autonomic Flexibility and Neurovisceral Integration models assert that an adaptive organism shows high dynamic variability in the cognitive, behavioral and physiological states that it occupies over time (Friedman and Thayer, 1998a, b; Thayer and Lane, 2000, 2009; Friedman, 2007). Here, “dynamic” describes variability on a gradient of situational demands, where psychophysiological responses vary to fit the diverse environmental challenges that characterize daily life (Beer, 1995). Such complex variability in responding is thought to be supported by cardiac vagal control, in part because vagal control dynamically regulates HR and thus metabolic output to fit rapidly changing environmental demands (see Polyvagal Theory; Porges, 1995a,b, 2007, 2009). In addition, vagal control is a suggested proxy for the inhibitory action of the prefrontal cortex (PFC), which sculpts motivational circuitry and hence cognitive-behavioral responses to fit varying challenges (Thayer and Lane, 2000, 2009; Thayer, 2006).

Much empirical research generated from these theories has focused on *tonic vagal control*, mean levels of vagal control measured as HF-HRV during rest. Tonic vagal control is theorized as a trait metric that indexes the organism’s capacity for context-appropriate responding across varied stressors (Porges, 1992, 1995a; Thayer and Lane, 2009). Supporting this notion, high tonic vagal control has been associated with decreased performance and greater defensive responding during intense or immediate threat (e.g., military survival training, threat with actual delivery of shock; Ruiz-Padial et al., 2003; Morgan et al., 2007).

Central to the aforementioned models of vagal control, tonic vagal control is thought to support phasic vagal control at the state level—manifested as IIV in vagal control between diverse challenges (Porges et al., 1994; Thayer and Lane, 2000). Relative to tonic vagal control, IIV in vagal control more directly represents active shifts in physiological activity (i.e., HR, PFC activity). Thus, greater variability in vagal control is believed to reflect more effective titration of physiological state to different environmental demands, which according to leading theories of vagal function, characterizes adaptability of the organism (Friedman and Thayer, 1998a, b; Thayer and Lane, 2000). Although tonic vagal control and IIV in vagal control work together to support adaptive responses under stress, IIV may also play a unique role in context-appropriate responding (Porges, 2007).

Current understanding of IIV in vagal control and its relationship to context-appropriate responding is limited because prior studies have examined IIV with difference scores reflecting change in HF-HRV between rest and a single stressor. Here, larger changes in HF-HRV have been associated with relatively higher resting HF-HRV (tonic vagal control; e.g., Porges et al., 1996; Salomon, 2005; Mathewson et al., 2010; Muhtadie et al., 2015; Rigoni et al., 2017), and greater context-appropriate cognition in the form of higher cognitive performance under low stress (Duschek et al., 2009; Mathewson et al., 2010; Elliot et al., 2011). These difference scores inherently reflect a single shift in state and therefore do not probe overall variability in vagal control across a gradient of environmental challenges—i.e., dynamic IIV that have been theorized to represent adaptability of the organism. A more direct empirical test of such notions requires a metric of IIV that better takes into account multiple and varied situations.

### Current Study

To better reflect dynamic IIV, we examined overall variability in vagal control between rest and multiple stressors, measured as the standard deviation (SD) of HF-HRV across four different conditions (rest, training, low stress, high stress). The aim of the current study was to examine theorized links between tonic vagal control (measured as resting HF-HRV), dynamic IIV in vagal control, and context-appropriate changes in response inhibition under stress. We focus on response inhibition because it is a central component of top-down cognition that is both affected by stress and strongly linked to vagal function (Thayer and Lane, 2009; Miyake and Friedman, 2012). Since tonic vagal control is theorized to support dynamic vagal regulation, we predicted that greater IIV in vagal control between conditions would be associated with higher levels of tonic vagal control. In accord with prior research, we predicted that higher tonic vagal control would be associated with adaptive responding to stress, reflected as stronger decreases in response inhibition due to high stress. Since high levels of such dynamic IIV are theorized to support shifts in cognitive-affective state (Thayer and Friedman, 1997; Friedman, 2007), we also hypothesized that individuals with greater variability in HF-HRV between conditions would show greater decreases in response inhibition due to high stress. We predicted that this effect would persist even when controlling for tonic vagal control, in line with a unique role for IIV in behavioral adaptability (Porges, 2007).

## Materials and Methods

### Subjects

Thirty-three United States Army Infantrymen or Special Reaction Team members (all men) participated in this study on a voluntary basis. Subjects were recruited from a pool of military personnel at Aberdeen Proving Ground and neighboring military installations, via email correspondence with previous research participants as well as research and training organizations within the U.S. Army. Subjects were excluded based on presence of: (i) a mood or anxiety disorder; (ii) cardiovascular illness; and (iii) cardiac pacemaker, as these factors may exacerbate the harmful consequences of the electric shock used in our task. Although women were included in recruitment, none volunteered. In order to ensure ecologically valid results, subjects were also required to meet the minimum Army specified requirements of marksmanship. This study was carried out in accordance with the recommendations of the Institutional Review Board at the U.S. Army Research Laboratory with written informed consent from all subjects. All subjects gave written informed consent in accordance with the Declaration of Helsinki. The protocol was approved by the Institutional Review Board at the U.S. Army Research Laboratory Human Research Protection Program (32 CFR 219 and DoDI 3216.01). Seven subjects were excluded due to technical malfunctions of the physiological recording equipment, and one additional outlier was excluded (see “Statistical Analyses” section). Consequently, 25 men were retained in our analyses (*Mean Age* = 30.73, *SD* = 7.71).

### Behavioral Task

Subjects completed a shooting-based target discrimination task that was implemented in the Army Research Laboratory Immersive Cognitive Readiness Simulator (ICoRS) with the VirTra V 300™ software. Here, an ecologically valid (Patton, 2014), 300-degree environment used five 6 × 10’ screens to simulate a quarry landscape where virtual human avatars intermittently appeared from behind objects. The weapon employed was a standard U.S. Army M-4 carbine rifle using CO_2_ in the ammunition magazines and an infrared laser that interfaced with the ICoRS system to give the subject feedback (Patton, 2014).

An auditory tone-oriented subjects to the position of the human avatars, which were always presented in pairs for a duration of 2 s. The two avatars could either be: (1) two “friends” (friend trial); or (2) one "friend” and one “foe” (foe trial). Foes were defined as avatars holding a black pistol, and friends as avatars holding non-threatening objects or holding their hands up. For each trial (i.e., single presentation of two avatars), subjects had 2 s to make a discrimination of friend or foe by firing a rifle at targets they identified as foes. Friendly trials required that shots at friends be withheld, while foe trials required that enemy targets be shot. For friendly trials, one could either make a false alarm (shooting a friend) or a correct rejection (CR; not shooting a friend). For foe trials, there could be either a hit (shooting a foe) or a miss (not shooting a foe). Subjects were instructed to hit as many foes and as few friendly targets as possible.

The discrimination task was broken into three conditions: training, vibration and shock. During the training condition, subjects practiced discriminating targets and firing the rifle. Incorrect decisions during training received no feedback. Subjects were presented with 128 avatar pairs (64 friend trials; 64 foe trials) in the shock and vibration conditions separately (see below), such that there were 256 experimental trials in total. Subjects received tactile feedback in response to incorrect responses. In the vibration condition, the feedback was a 500 ms vibration delivered to one side of the waist by the VirTra Threatfire™. In the shock condition, feedback was an aversive 200 ms, 50 mA electric shock using the VirTra Threatfire™ shock belt. Shocks were delivered on a single side of the waist, with the side of administration alternating with each shock. Performance-dependent electric shock is conceptualized as a threat that elicits stress, involving increased subjective reports of negative emotion, heightened cardiovascular reactivity (Light and Obrist, 1980; Keinan, 1987). Therefore, we conceptualize the shock and vibration conditions as conditions of high stress and low stress, respectively.

### Procedure

**Figure 1:**
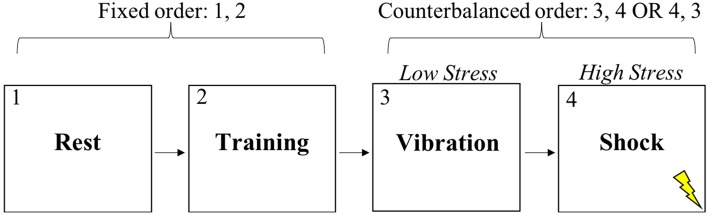
Experimental task conditions.

After providing informed consent, subjects completed a 10-min resting baseline while sitting in a quiet, dimly lit room (see Figure 1). After the experimenter explained the nature of the shooting task, subjects completed practice trials until they verbally indicated to the experimenter that they understood the task. Next, subjects completed the training condition of the shooting task which had a duration of approximately 9 min. No instructions were provided to the subject during training. The resting period and training were in the same order for all subjects. Subjects then completed either the low stress or high stress condition of the discrimination task, each of which lasted approximately 17 min. The order of the low and high stress conditions was randomly counterbalanced across subjects. Subjects were standing for all experimental conditions other than the resting baseline. In all, subjects experienced four experimental conditions during which vagal control and respiration rate (RR) were continuously measured.

This study was carried out in accordance with the recommendations of the Institutional Review Board at the U.S. Army Research Laboratory with written informed consent from all subjects. All subjects gave written informed consent in accordance with the Declaration of Helsinki. The protocol was approved by the Institutional Review Board at the U.S. Army Research Laboratory.

### Measures

#### Behavioral

##### Response Inhibition

The first shot fired in each trial was used to compute performance. Prior to computing performance, trials were excluded if subjects oriented to the wrong screen at trial onset. In the analyses, we focus on CRs of friendly targets as a metric of successful response inhibition. The proportion of CRs was defined as the number of CRs out of the total number of friendly trials.

##### Effect of Stress on Response Inhibition

The effect of stress on response inhibition was computed with a difference score that subtracted the proportion of CRs during low stress from the CRs during the high stress condition (CR high stress − CR low stress). A score was computed for each person, and larger negative values indicated stronger decreases in response inhibition due to stress.

#### Physiological

##### HRV and Respiration Quantification

Electrocardiography (ECG) and RR were continuously assessed throughout the experiment with the Q02 Equivital™ LifeMonitor sensor belt worn across the thorax (Equivital, Hidalgo, UK). ECG was collected from two electrodes on the chest and another below the clavicle, while RR was captured with a pressure transducer at the sternum using in the same device. ECG and respiration signals were cleaned and analyzed offline with Vivosense^®^ software (Vivonoetics, San Diego, CA, USA). Artifact correction was applied to the interbeat interval (IBI) time series with high noise filtering and removal of ectopic beats (IBIs <300 ms or >1,500 ms). The cleaned IBI series was interpolated and resampled at 4 Hz and then submitted to fast Fourier transform (FFT) spectral analysis to compute HF-HRV in the 0.15–0.4 Hz band (Malik et al., 1996) as our measure of cardiac vagal control (Malliani et al., 1994). HF-HRV values were computed in absolute units (ms^2^). Separate spectral estimates of HF-HRV were computed for each condition, which yielded four HF-HRV estimates for each subject. After artifact correction and smoothing of the respiration signal in Vivosense software, RR estimates were quantified for each condition.

##### IIV and Tonic Vagal Control

Due to their skew, HF-HRV metrics were normalized with a natural logarithm, and these transformed metrics are denoted as lnHF-HRV. IIV in vagal control was computed for each subject as the SD of the four condition-level lnHF-HRV values (rest, training, low stress, high stress). Higher SDs indicated that a subject had greater differences in HF-HRV between the conditions overall. In line with prior research, tonic vagal control was represented with resting lnHF-HRV^1^.

### Statistical Analyses

One outlier (>3 SD) on our primary performance metric (CR high stress − CR low stress) was removed, leaving 25 subjects in all analyses and results. The distributions of variables were confirmed to be normal based on Spiegelhalter’s (1980) omnibus tests of normality for small samples. Nonlinearity in HRV-performance relationships were visually screened, which indicated that all associations were strictly linear. In order to test if stress impaired response inhibition, we used a paired *t*-test that contrasted CRs between low and high stress conditions. Pearson correlations (*df* = 23) tested hypothesized relationships among lnHF-HRV metrics and stress-related inhibition performance (CR high stress − CR low stress). To test whether IIV uniquely related to stress-related performance apart from tonic vagal control, partial Pearson correlation coefficients (PCC) examined the relationship between the SD of lnHF-HRV (i.e., IIV) and performance while adjusting for tonic vagal control (i.e., resting lnHF-HRV). We tested all effects with two-tailed *p*-values and an alpha of 0.05. Associations were also interpreted based on effect size (Wilkinson, 1999). We also examined correlations between lnHF-HRV metrics and the difference in the proportion of hits between low and high stress. None of these effects were significant (*p* < 0.05; α = 0.05) and are thus not reported here.

## Results

### Manipulation Check

Subjects made a lower proportion of CRs during high (Mean = 0.84, SD = 0.09) relative to low (Mean = 0.88, SD = 0.09) stress conditions, *paired t*_(24)_ = −3.45, *p* = 0.002.

### Relationships Between Vagal Control Metrics

See Table 1 for the correlations among physiological and performance metrics. The SD of lnHF-HRV was positively associated with resting lnHF-HRV, a common metric of tonic vagal control (*r* = 0.43, *p* = 0.031).

**Table 1:** Descriptive statistics and zero-order correlations (*n* = 25).

	Mean (SD)	1	2	3	4	5	6	7
1. Resting lnHF-HRV (ln(ms^2^))	1.88 (0.31)	1						
2. SD of lnHF-HRV (ln(ms^2^))	0.47 (0.33)	0.43*	1					
3. Resting RR (bpm)	8.13 (3.23)	−0.21	−0.09	1				
4. SD RR (ln(bpm))	3.54 (1.78)	−0.04	0.12	−0.60**	0.20	1		
5. High stress-CR (# CR/total)	0.84 (0.09)	−0.18	−0.21	−0.16	−0.05	0.19	1	
6. Low stress-CR (# CR/total)	0.88 (0.09)	−0.11	0.07	−0.20	0.003	0.33^†^	0.80***	1
7. CR (high stress— low stress)	−0.04 (0.06)	−0.13	−0.45*	0.03	−0.09	−0.20	0.44*	−0.18

Notes. HF-HRV, high-frequency heart rate variability; ln, natural logarithm; SD, standard deviation; RR, respiration rate; bpm, breaths per minute; CRs, correct rejections (proportion of friendly trials with CRs); CR (high stress − low stress) = (proportion of CRs in high stress) − (proportion of CRs in low stress). Two-tailed p-values ^†^p < 0.10, *p < 0.05, **p < 0.01, ***p < 0.001.

### Vagal Control and the Effect on Stress on Response Inhibition

#### Tonic Vagal Control

Resting lnHF-HRV (*r* = −0.13, *p* = 0.537) was not significantly associated with the difference in CRs between low and high stress (Table 1). When adjusting for resting RR, the effect size of the association between resting lnHF-HRV and performance (*PCC* = −0.24, *p* = 0.274) became larger.

#### IIV in Vagal Control

**Figure 2:**
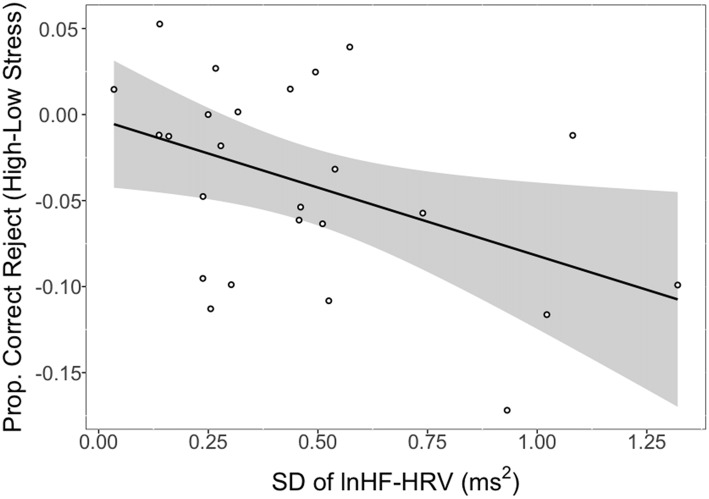
Proportion of correct rejections (CRs) in high stress (shock) vs. low stress (vibration) as a function of intra-individual variability (IIV) in natural logarithm transformed high-frequency heart rate variability (lnHF-HRV; standard deviation (SD) of lnHF-HRV). *Notes*: line reflects a significant association between the SD of lnHF-HRV and CRs (high stress − low stress). Shaded region represents the 95% confidence interval. SD of lnHF-HRV reflects the SD of condition-level lnHF-HRV values (rest, training, vibration, shock). Larger negative values on the *y*-axis indicate stronger impairing effects of stress on response inhibition. Larger positive values on the *x*-axis represent greater IIV in vagal control.

There was a significant association between the SD of lnHF-HRV and the difference in CRs between low and high stress (*r* = −0.45, *p* = 0.026). This relationship is depicted in Figure 2 where larger SDs in lnHF-HRV were associated with a lower proportion of CRs in high relative to low stress. Conversely, smaller SDs in lnHF-HRV were associated with a relatively comparable proportion of CRs between low and high stress. In other terms, greater differences in vagal control between the conditions (rest, training, low stress and high stress) were related to stronger impairments in response inhibition due to stress.

To examine if this IIV relationship is unique from tonic vagal control, we examined the correlation between the SD of lnHF-HRV and stress-related performance (i.e., CR high stress − CR low stress) while controlling for resting lnHF-HRV. The correlation between the SD and stress-related performance remained significant (*PCC* = −0.44, *p* = 0.033)^2^.

## Discussion

As hypothesized, individuals with high tonic vagal control exhibited stronger stress-related reductions in response inhibition performance, in that the size of this relationship mirrored previous research (e.g., Hansen et al., 2003; Kaufmann et al., 2012). Also as hypothesized, individuals with greater differences in vagal control between the task conditions (higher IIV) evidenced higher tonic vagal control, and, importantly, stronger decreases in response inhibition performance due to stress. The relationship between IIV and performance remained when statistically controlling for tonic vagal control, suggesting that tonic and phasic vagal function work together to promote adaptive changes in cognition during stress. Findings also underscore dynamic IIV across multiple, varied conditions as a potentially unique aspect of vagal function that is important for context-appropriate responding and adaptability.

### Tonic Vagal Control and Response Inhibition Under Stress

The direction of the relationship between tonic vagal control at rest and stress-related performance is consistent with a prior study in which resting vagal control was inversely correlated with performance requiring cognitive control (Morgan et al., 2007). Importantly, these effects are in the opposite direction of other studies that detected positive associations between resting vagal control and performance (e.g., Hansen et al., 2003, 2009; Johnsen et al., 2003; Hovland et al., 2012). Unlike these prior studies, the ecologically valid stressors used here (immersive shooting environment) and in Morgan et al. (2007) (military survival training) may have represented contexts where decreased inhibition was adaptive for survival and supported by high tonic vagal control and greater IIV in vagal control (see below). That is, we observed decreases in response inhibition during an ecologically valid and salient threat (see “Manipulation Check” in results section; Keinan et al., 1999). Such decreases in inhibition are conceptualized as an adaptive response that promotes defensive responses and survival (Keinan, 1987; Nesse, 2005; Lang and Davis, 2006).

It should also be noted that the relationship between resting vagal control and performance was consistent with prior research only when correcting for respiration. This finding may indicate links between tonic vagal control and cognition apart from phasic respiratory modulation of cardiac vagal control (Houtveen et al., 2002).

### IIV in Vagal Control: Associations With Tonic Vagal Control and Response Inhibition

Unlike prior work, which has operationalized IIV as reactivity between rest and a single stressor (e.g., Porges et al., 1996; Salomon, 2005; Mathewson et al., 2010), the current study investigated overall variability in vagal control on a gradient of challenges (rest, training, low stress, high stress). Our approach better captures the ability of the organisms to modulate psychophysiological responses, not only from rest to stress, but also between different stressors. Such dynamic variability is the hallmark of an adaptive organism in leading models of vagal function (Autonomic Flexibility, Neurovisceral Integration, Polyvagal Theory; Porges, 1995b; Thayer and Friedman, 1997, 2002; Friedman and Thayer, 1998a, b; Thayer and Lane, 2000, 2009). As a consequence, our findings are among the first to support theorized interrelations between *dynamic* variability in vagal control, tonic vagal control, and context-appropriate cognition under stress.

The present relationship between high resting vagal control and greater IIV expand on prior associations between tonic vagal control and vagal reactivity (stress-rest; Salomon, 2005; Muhtadie et al., 2015). By utilizing an index of overall variability, our findings more directly support tonic vagal control as a trait that modulates phasic vagal responses across a wide variety of cognitive-affective demands (Thayer, 2006; Friedman, 2007; Porges, 2007). Importantly, the present relation between IIV and stress-related response inhibition suggests that greater shifts in vagal control between challenges may in part characterize the organism’s ability to exhibit context-appropriate changes in cognition. This inference is in line with models of vagal function that emphasize dynamic IIV (Thayer and Lane, 2000). Interpreting organismic functioning with a dynamical systems view, these findings suggest that complex, broad-scale variability in vagal function permits efficient entry into a state of high stress. Such high stress is accompanied by an adaptive disinhibition of defensive responses (impaired response inhibition) to cope with threat (Nesse, 2005; Deco et al., 2011; Garrett et al., 2013; Oken et al., 2015).

The present intercorrelations among resting vagal control, IIV, and performance confirm that dynamic IIV in vagal control works alongside tonic vagal control to promote context-appropriate cognition under stress (Porges et al., 1996; Thayer and Lane, 2000).

Nevertheless, the present findings also support a relationship between IIV and response inhibition that is unique from tonic vagal control. These results suggest separable neural mechanisms for tonic and phasic vagal control that have been explicated in prior work. In Polyvagal Theory, dynamic modulation of the vagal brake better indexes the active neural mechanisms of behavioral regulation than does tonic vagal control (Porges, 1995b, 2007). Similarly, in the Neurovisceral Integration Model (Thayer and Lane, 2000, 2009), IIV in vagal control reflects phasic changes in ventromedial PFC (vmPFC) activity (Lane et al., 2010) while tonic vagal control reflects the tonic inhibitory output of the vmPFC on motivational circuitry (Thayer et al., 2012).

In contrast to our study, some studies report that vagal control is related to cognition at the tonic but not phasic level (e.g., Laborde et al., 2015; Spangler et al., 2015). This suggests that the relative contributions of tonic and phasic (IIV) vagal control to cognitive adaptability depend on factors that varied between these studies (e.g., sample, experimental context). Namely, examination of IIV in vagal control may require that researchers utilize robust conditions capable of eliciting sufficient parasympathetic reactivity (Overbeek et al., 2012, 2014).

## Limitations, Future Directions and Conclusions

Despite a number of strengths (measurement of HF-HRV across varied task demands, control for respiratory variability, ecologically valid task), the current study also has limitations. The present results might only apply to military personnel, and associations in civilians may differ due to their lack of training and less exposure to high stress. Also, the present results might be limited to men, as there were no women in the current sample. Sex differences in vagal function have been noted, suggesting sex differences in IIV-performance relationships as a fruitful avenue for future research (Koenig and Thayer, 2016). Furthermore, it may be adaptive to preserve response inhibition under demands less stressful than those used in the current study, making high dynamic IIV relate to *less* impaired response inhibition in such low stress scenarios. Future studies should investigate IIV in vagal control in different samples and contexts to see if IIV-performance relations differ between: (i) military and civilian populations; (ii) men and women; and (iii) types of stress. The present study is also limited in its ability to precisely characterize the specific patterns of IIV in vagal control and their psychophysiological mechanisms. Additional research with a larger sample size is required to statistically model the trajectories of HF-HRV change across conditions. Additional studies might also investigate how IIV is systematically related to task demands (social, emotional, cognitive) that are theoretically related to vagal control (Jennings, 1992; Porges, 2007; Thayer and Lane, 2009). This study was also limited in that it did not measure sympathetic nervous system activity, an important autonomic contribution to cardiac responses under stress (Cannon, 1932). Future work should utilize cardiac sympathetic metrics from impedance cardiography to more comprehensively study the autonomic concomitants of response inhibition under stress. Lastly, the current study did not account for critical covariates that are relevant to cognition (e.g., IQ) or emotion (e.g., anxiety, depression) which might confound relations between vagal control and response inhibition. Further research is therefore needed to examine the influence of such factors on the current findings.

In sum, our findings provide preliminary evidence that IIV in vagal control across multiple conditions predicts stress-related effects on cognition among men in a military context. They also illustrate the utility of considering variability of vagal function beyond metrics of tonic vagal control. Additionally, our results inform a potential index of the neural mechanisms underlying stress and cognition, which could ultimately be leveraged in operational settings to improve human performance. In particular, the present findings provide preliminary evidence that cardiac vagal metrics are useful for monitoring real-world performance outcomes in military personnel.

## Author Contributions

DS performed primary data analyses and writing responsibilities. KG and JM helped in writing the manuscript. Both JB and JT assisted in directing the vision of the article and consulted on the statistical analyses.

## Conflict of Interest Statement

The authors declare that the research was conducted in the absence of any commercial or financial relationships that could be construed as a potential conflict of interest.

## Footnotes

^1^Respiration metrics were uncorrelated with HF-HRV and performance metrics. Therefore, we report correlations between HF-HRV and stress-related performance that are not corrected for RR. All correlations remained the same (*p* < 0.05) when adjusting for respiration, unless otherwise noted (see “Tonic Vagal Control” section).

^2^The correlation between the SD of lnHF-HRV and performance might also be confounded by mean vagal control across the conditions, since relationships involving IIV are often driven by the intra-individual average on the same variable (Hultsch et al., 2008; Segerstrom et al., 2017). Consequently, the mean of condition-level lnHF-HRV values was computed as a covariate. The correlation between the SD of lnHF-HRV and stress-related CRs remained significant when controlling for mean lnHF-HRV (*PCC* = −0.43, *p* = 0.036).
